# Re-evaluation of single nucleotide variants and identification of structural variants in a cohort of 45 sudden unexplained death cases

**DOI:** 10.1007/s00414-021-02580-5

**Published:** 2021-04-25

**Authors:** Jacqueline Neubauer, Shouyu Wang, Giancarlo Russo, Cordula Haas

**Affiliations:** 1 Zurich Institute of Forensic Medicine, University of Zurich, Zurich, Switzerland; 2 Department of Forensic Medicine, School of Basic Medical Sciences, Fudan University, Shanghai, China; 3 Functional Genomics Center Zurich (FGCZ), University of Zurich/ETH, Zurich, Switzerland

**Keywords:** Forensics, Massively parallel sequencing (MPS), Exome sequencing, Single nucleotide variant (SNV), Structural variants (SV), Copy number variation (CNV)

## Abstract

Sudden unexplained death (SUD) takes up a considerable part in overall sudden death cases, especially in adolescents and young adults. During the past decade, many channelopathy- and cardiomyopathy-associated single nucleotide variants (SNVs) have been identified in SUD studies by means of postmortem molecular autopsy, yet the number of cases that remain inconclusive is still high. Recent studies had suggested that structural variants (SVs) might play an important role in SUD, but there is no consensus on the impact of SVs on inherited cardiac diseases. In this study, we searched for potentially pathogenic SVs in 244 genes associated with cardiac diseases. Whole-exome sequencing and appropriate data analysis were performed in 45 SUD cases. Re-analysis of the exome data according to the current ACMG guidelines identified 14 pathogenic or likely pathogenic variants in 10 (22.2%) out of the 45 SUD cases, whereof 2 (4.4%) individuals had variants with likely functional effects in the channelopathy-associated genes *SCN5A* and *TRDN* and 1 (2.2%) individual in the cardiomyopathy-associated gene *DTNA*. In addition, 18 structural variants (SVs) were identified in 15 out of the 45 individuals. Two SVs with likely functional impairment were found in the coding regions of *PDSS2* and *TRPM4* in 2 SUD cases (4.4%). Both were identified as heterozygous deletions, which were confirmed by multiplex ligation-dependent probe amplification. In conclusion, our findings support that SVs could contribute to the pathology of the sudden death event in some of the cases and therefore should be investigated on a routine basis in suspected SUD cases.

**Supplementary Information:**

The online version contains supplementary material available at 10.1007/s00414-021-02580-5.

## Introduction

Sudden death events in young individuals often represent the first manifestation of an undetected genetic disease, which remained without any symptoms during lifetime. Although some of the deceased show cardiac structural abnormalities and can be explained by autopsy, approximately 30% of sudden death cases still remain elusive and are therefore termed as sudden unexplained death (SUD) [1, 2]. In the past years, postmortem molecular autopsy enabled the identification of hidden cardiac diseases in a series of SUD cases. These diseases include mainly channelopathies or cardiomyopathies associated with single nucleotide variants (SNVs) located in the coding regions of functional genes [3–6]. In addition, some variants located in noncoding regions, such as regulatory elements and splice sites, might be accountable for serious functional effects [7–10]. Recent studies revealed that structural variants (SVs), including copy number variations (CNVs), might play an important role in SUD and could explain some of the unresolved cases [11–14].

SVs include diverse genomic alterations like deletions, duplications, insertions, inversions, translocations, or complex rearrangements of relatively large segments such as tandem exon duplications, or complex gains or losses of homologues sequences at multiple sites in the genome, commonly referred to as CNVs [15, 16]. While SVs are considerably less common than SNVs, they have greater functional potential due to their larger size, and they are more likely to alter gene structure [17]. SVs and CNVs can influence gene expression, phenotypic variation, and adaptation, by disrupting genes and altering gene dosage. Many SVs are described to confer risk to complex disease traits or increase disease susceptibility to e.g. HIV-1 infection [18] or neurodevelopmental disorders including autism [19], schizophrenia [20], and depression [21]. Besides, pathogenic CNVs have been reported in cardiac diseases such as Brugada syndrome (BrS) [11], arrhythmogenic right ventricular cardiomyopathy (ARVC) [12], or sudden cardiac death (SCD)-related cases [13, 14]. One previous study examined CNVs in a large cohort of SUD cases and patients that had an inherited cardiac disease and discovered that the frequency of identified CNVs varied from 1.4 to 5.1% among cases with different underlying diseases [22]. Another study investigated a small cohort of 13 sudden arrhythmic death syndrome (SADS) and sudden unexplained death in infancy (SUDI) cases with whole genome sequencing and whole transcriptome sequencing. However, they did not find any SVs in the coding and regulatory regions of 100 cardiac genes [23]. A recent study focused on 2 large Amish families with multiple sudden deaths and sudden cardiac arrests in young individuals and identified a homozygous multi-exon duplication in *RYR2* [24]. Another study investigated a Tunisian family with high incidence of SCD in young family members, but did not find any pathological relevant CNVs [25]. The lack of a consensus of the impact of SVs on inherited cardiac diseases emphasized the need of more evidence on this topic.

The aim of this study was to screen for potentially pathogenic SVs in 244 genes associated with cardiac diseases in a cohort of 45 SUD cases. Whole-exome sequencing (WES) in 35 out of these 45 cases has already been performed earlier [4], whereas 10 additional SUD cases were analyzed as part of this study. Due to updates in the recommendations for rare variant classification, the exome sequencing data of all 45 SUD cases was analyzed and classified according to the current ACMG guidelines [26].

## Materials and methods

### SUD cohort

All SUD cases had been autopsied at the Zurich Institute of Forensic Medicine (ZIFM) in Switzerland between 2012 and 2019. The sudden death victims were examined according to the respective European and Swiss guidelines for the management of young SUD cases [27–29], including a thorough death scene investigation, a complete autopsy with pharmacological-toxicological and histopathological screening, and a review of the clinical history. The SUD cohort consisted of 45 cases with a mean age of 30.2 ± 14.5 years (range: 1–63 years of age) (Table S1). Seventy-five percent of the deceased were males and most of them were of European origin (89%). Another 5 cases (11.1%) were of African (3 cases), Indian (1 case), and Chinese (1 case) origin. Exome sequencing data was already available for 35 of the SUD cases [4]. Additional 10 SUD cases were analyzed according to the workflow described in our previous study [4].

### Exome sequencing and bioinformatics

Genomic DNA of the 10 new SUD cases was obtained from shock-frozen kidney tissue. DNA extraction was performed using the QIAamp DNA Mini Kit (Qiagen, Hombrechtikon, Switzerland) according to the manufacturer’s protocol. DNA quantities were determined with a Quantus™ Fluorometer (Promega, Dübendorf, Switzerland). The SureSelect^XT^ target enrichment and SureSelect^XT^ All Exon V5 + UTR kits (Agilent Technologies AG, Basel, Switzerland) were used for DNA library preparation and exome capture, followed by quality and quantity assessment with the TapeStation system using DNA 1000 ScreenTapes (Agilent Technologies AG). DNA libraries were sequenced on an Illumina NovaSeq 6000 instrument (Illumina Inc., San Diego, CA, USA) with 150 paired-end reads and a mean coverage of 65 × per sample. Sequences were aligned to the reference genome (GRCh37/hg19) using BWA [30] and samples were required to have at least 80% of the exome covered at ≥ 20 × read depth. Variant discovery was performed by means of GATK [31], following the GATK best practice workflow [32].

### SNV identification

Data analysis was confined to a target gene panel consisting of 244 genes associated with cardiac diseases (Table S2). The annotation of the VCF files was performed with the software Alamut Batch, version 1.11 (Interactive Biosoftware, Rouen, France) and the visualization of the coverage of SNVs was done with the software IGV, version 2.4.16 [33]. SNV screening was performed according to an updated version of our previously published in-house filtering strategy [4] and to the ACMG standards and guidelines for the interpretation of sequence variants [26]. Our filter strategy selects SNVs with a stringent global minor allele frequency value (MAF) of less than or equal to 0.005% derived from the genome aggregation database (gnomAD) which is the largest available human database, including 125,748 exomes and 15,708 whole genomes from unrelated individuals of different ethnicities, sequenced as part of various population genetic studies [34]. In addition, synonymous and intronic variants were excluded, focusing only on exonic and splice site SNVs. Pathogenicity of the SNVs was assigned based on the evaluation of variant types (null variants, frameshift variants, splice site variants, or missense variants) and on the genome interpretation database VarSome [35]. Identified SNVs have been submitted to the Leiden Open Variation Database (Individuals Nr. 00,315,499–00,315,504 and 00,324,655–00,324,658) (http://databases.lovd.nl).

### SV identification

SVs were called using LUMPY [15]. Briefly, discordant paired-end alignments were retrieved using samtools [36] (*samtools view -b -F 1294*) and the split-read alignments were extracted using the LUMPY auxiliary script *extractSplitReads_BwaMem*. The discordant paired-end and the split-read file were then parsed, together with the alignment (bam) file to *lumpyexpress* with default settings. The resulting VCF files were annotated using ANNOVAR [37] using the UCSC human genome version GRCh37/hg19 as reference and the Database of Genomic Variants (DGV) as variants database [38]. Among the SVs within our target genes, those identified in more than one sample were excluded from subsequent analysis, since pathogenic SVs that contribute to SUD should be rare variants according to the ACMG guidelines [26]. Furthermore, SVs longer than 1 Mbp were excluded because in these cases, the expression levels of too many genes were considered abnormal, which makes them difficult to validate. The remaining SVs were manually checked using the IGV software; only those with a characteristic gene expression alteration were considered eligible SV candidates. Allele frequencies of the candidate SVs were checked in the gnomAD-SV database [45].

### MLPA validation

The identified candidate SVs were confirmed by multiplex ligation-dependent probe amplification (MLPA) according to the manufacturer’s instructions. Self-designed oligos for the detection of our target regions were synthesized by Microsynth (Microsynth AG, Balgach, Switzerland). In brief, 100 ng of DNA and a customized probemix consisting of synthetic oligos (Table S3) and the P200 reference probemix (MRC Holland, Amsterdam, Netherlands) were used in the hybridization reaction. Fragment separation was performed on a 3130xl Genetic Analyzer (Thermo Fisher Scientific, Reinach, Switzerland). Fragment analysis and comparative analysis results were generated using Coffalyser.Net software (MRC Holland).

## Results

### SNV identification

Following the current ACMG guidelines for variant interpretation [26], 14 pathogenic or likely pathogenic SNVs were identified in 10 (22.2%) out of the 45 SUD cases (Table 1 and Table S4). These variants were located in genes that are linked to cardiomyopathies (1 SUD case), ion channelopathies (2 SUD cases), connective tissue diseases and/or congenital malformation syndromes (3 SUD cases), and metabolic diseases (4 SUD cases). Out of these 14 SNVs, 2 heterozygous stop-gain variants (*DTNA*, NM_001390.4, c.2224C > T, p.(Gln742*) and *LZTR1*, NM_006767.4, c.2440C > T, p.(Gln814*)), 1 heterozygous two-base duplication (*CALR3*, NM_145046.4, c.387dup, p.(Ile130Tyrfs*11)), 1 homozygous one-base deletion (*LZTR1*, NM_006767.4, c.604_605del, p.(Met202Valfs*57)), and 1 heterozygous missense variant (*SCN5A*, NM_001099404.1, c.2204C > A, p.(Ala735Glu)) were predicted as pathogenic. In addition, 2 heterozygous stop-gain variants (*ALMS1*, ENST00000264448.6, c.54_55insTAG, p.(Glu19*) and *MLYCD*, NM_012213.3, c.1073C > A, p.(Ser358*)), 2 heterozygous deletions (*SLC37A4*, ENST00000357590.5, c.528del, p.(Val177Trpfs*35) and *TRDN*, NM_006073.4, c.1923_1924del, p.(Leu643Serfs*19)), and 5 heterozygous missense variants (*ACADS*, NM_000017.4, c.320G > A, p.(Arg107His); *ACADS*, NM_000017.4, c.814C > T, p.(Arg272Cys); *SOS1*, NM_005633.3, c.2728G > T, p.(Asp910Tyr); *FBN2*, NM_001999.4, c.3437A > G, p.(Tyr1146Cys) and *ANK2*, NM_001148.5, c.4158C > G, p.(Phe1386Leu)) were predicted as likely pathogenic.

**Table 1:**
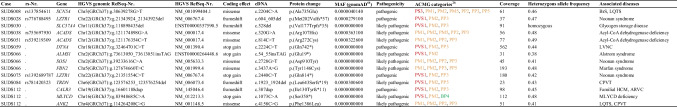
Pathogenic or likely pathogenic SNVs identified in our SUD cohort

*ACMG* American College of Medical Genetics and Genomics recommendations, *ARVC* arrhythmogenic right ventricular cardiomyopathy, *BrS* Brugada syndrome, *CPVT* catecholaminergic polymorphic ventricular tachycardia, *gnomAD* genome aggregation database, *HCM* hypertrophic cardiomyopathy, *LQTS* long QT syndrome, *LVNC* left ventricular non-compaction cardiomyopathy, *NA* not available

Color description for ACMG categories: red = very strong and strong evidence of pathogenicity, orange = moderate and supporting evidence of pathogenicity, green = supporting evidence of benign impact

In general, the re-analysis and re-classification of our recently published exome data of the 35 SUD cases [4] caused a change in the classification of the pathogenicity in the previously reported 11 variants (Table S5). Six of the 11 previously reported variants in the genes *ACAD9* (p.(Arg420Cys)), *AKAP9* (p.(Asn2045Ser)), *FBN2* (p.(Gln2432His)), *MYLK* (p.(Arg1250His)), *SEMA3A* (p.(Arg66Trp)), and *RYR2* (p.(Glu1127Gly)) had a MAF greater than 0.005% and were therefore filtered out. The pathogenicity of the remaining 5 variants were down-classified from probably pathogenic (*BMPR2* (p.(Pro864Leu)) and *KCN5E* (p.(Tyr62Asn))) or likely pathogenic (*EFEMP2* (p.(Arg185His)), *RYR2* (p.(Ala3814Val)), and *RYR2* (p.(Gln4164Glu)) to uncertain significance (Table S5).

### SV identification

After pre-screening and IGV check, a total of 18 SVs were identified in 15 out of the 45 SUD cases (Table 2), located in 17 different genes (*ABCC9*, *CDH2*, *DMPK*, *DPP6*, *EFEMP2*, *FXN*, *GPD1L*, *KCNJ2*, *LAMA4*, *NOS1AP*, *PDLIM3*, *PDSS2*, *PPA2*, *PRKAG2*, *PRKG1*, *PTPN11*, *TRPM4*). The lengths of these SVs varied from 35 to 6079 bp, and most of them were positioned in the intergenic or intronic regions of our target genes. Out of these 18 SVs, the most promising 2 were heterozygous deletions located in the coding regions of *PDSS2* and *TRPM4*, respectively (Fig. S1).

**Table 2:** SVs identified in our SUD cohort

Case No.	Gene	Chrom.	Start position	End position	SV type	SV length (ABS)	Annotation	MAF (gnomAD-SV^44^)	Pathogenicity
SUDS006	*PRKG1*	chr10	53,578,486	53,578,799	DEL	313	Intronic in all gene isoforms	0.0000	Uncertain significance
SUDS021	*EFEMP2*	chr11	65,642,111	65,643,519	DEL	1408	Intergenic	0.5326	NA
SUDS023	*LAMA4*	chr6	112,430,849	112,431,010	DEL	161	Intronic in all gene isoforms	0.0000	Uncertain significance
SUDS028	*GPD1L*	chr3	32,102,052	32,107,883	DEL	5831	Intergenic	0.4791	NA
SUDS030	*DMPK*	chr19	46,278,659	46,279,615	DEL	956	Intronic in all gene isoforms	0.3946	Uncertain significance
SUDS030	*PDLIM3*	chr4	186,441,637	186,444,073	DEL	2436	Intronic in all gene isoforms	0.0000	NA
SUDS033	*KCNJ2*	chr17	68,455,097	68,461,176	DEL	6079	Intergenic	0.0000	NA
SUDS049	*ABCC9*	chr12	22,016,217	22,016,262	DEL	45	Intronic in all gene isoforms	0.0000	Uncertain significance
**SUDS058**	***PDSS2***	**chr6**	**107,519,092**	**107,519,163**	**DEL**	**71**	**Coding region (ENST00000449027)**	**0.0075**	**Uncertain significance**
SUDS067	*PPA2*	chr4	106,370,093	106,370,417	DEL	324	Intronic in all gene isoforms	0.0000	Uncertain significance
SUDS074	*PTPN11*	chr12	112,913,870	112,914,196	DEL	326	Intronic in all gene isoforms	0.0000	Uncertain significance
SUDS074	*PRKG1*	chr10	53,341,595	53,341,937	DEL	342	Intronic in all gene isoforms	0.0000	Uncertain significance
**SUDS075**	***TRPM4***	**chr19**	**49,686,029**	**49,686,064**	**DEL**	**35**	**Exonic in all gene isoforms**	**0.0000**	**Pathogenic**
SUDS075	*NOS1AP*	chr1	162,052,680	162,052,985	DEL	305	Intronic in all gene isoforms	0.0000	Uncertain significance
SUDS077	*CDH2*	chr18	27,629,690	27,630,006	DEL	316	Intergenic	0.5876	Uncertain significance
SUDS080	*PRKAG2*	chr7	151,523,113	151,523,386	DEL	273	Intronic in all gene isoforms	0.0000	Uncertain significance
SUDS085	*DPP6*	chr7	154,671,008	154,671,313	DEL	305	Intronic in all gene isoforms	0.0000	Uncertain significance
SUDS112	*FXN*	chr9	71,665,339	71,665,557	DEL	218	Intronic in all gene isoforms	0.0000	Uncertain significance

*ABS* absolute value*, DEL* deletion, *SV* structural variant, *MAF* minor allele frequency, *NA* pathogenicity not available in the human genomics database VarSome

The 71-bp deletion of *PDSS2* (NM_020381.4: c.1009-4103_1009-4033del), which can only be identified in exon 3 of one gene isoform (transcript ID: ENST00000449027), was predicted to be a variant with uncertain significance. This SV was identified in an 11-year-old previously healthy girl, who was swimming in the lake when she suddenly disappeared in the water. A lifeguard observed the incident and rescued her and immediately started resuscitation. The emergency team diagnosed a ventricular fibrillation and she became defibrillated for a single event. In the children’s hospital, she had a serious derailment of the acid–base balance and the sugar metabolism and died shortly after. According to the forensic investigations, the girl was already unconscious when she disappeared under water. Histological examination of the heart tissue revealed pre-existing changes in the excitation conduction system of the heart in the area of the secondary pacemaker center of the heart, which could have triggered cardiac arrhythmia and unconsciousness leading to a cardiovascular arrest.

The 35-bp deletion in exon 11 (transcript ID: ENST00000252826) of *TRPM4* (NM_017636.4: c.1459_1494del, p.(Lys487_Leu498del)) was predicted as pathogenic. This SV was detected in a 38-year-old male. He complained of discomfort after drinking some alcohol and was found dead some hours later in his bed. He did not have any medical history; however, his wife reported that he felt unusually tired and exhausted in the last 2 months prior to death. Autopsy investigation revealed an enlarged heart (520 g, 56% enlarged according to Zeek [40]) with thickening of the heart chamber wall muscle.

### MLPA validation

**Fig. 1:**
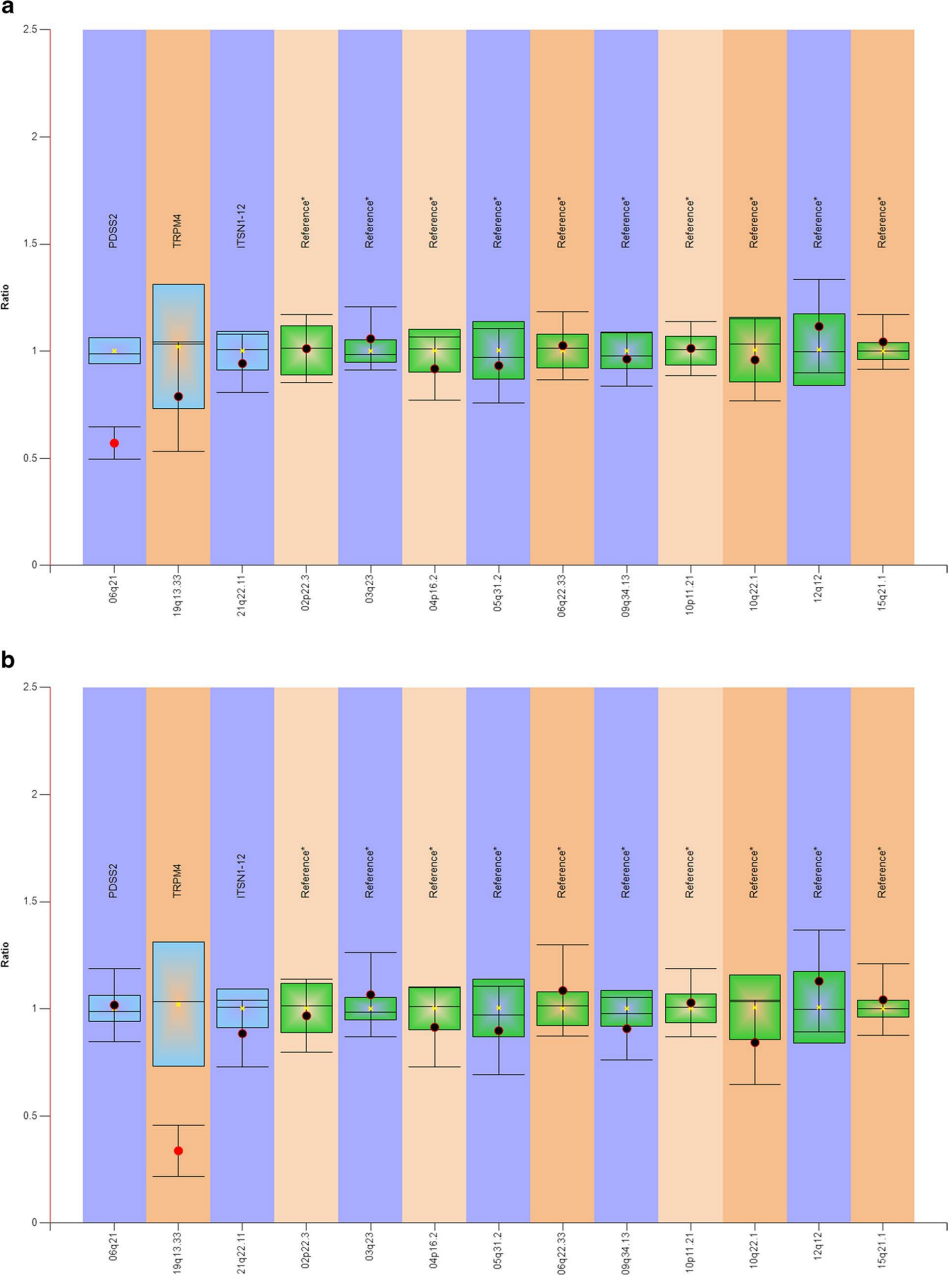
SVs confirmed by MLPA. Blue/green bars represent 95% confidence interval over the reference samples (*N* = 3), and dots with lines represent 95% confidence interval estimate for each probe. In our case, the 95% confidence intervals of **a** *PDSS2* in SUD058 and **b** *TRPM4* in SUD075 did not overlap, which suggests a heterozygous deletion

MLPA validation was performed on the 2 most promising SVs in the coding regions of *PDSS2* and *TRPM4*, respectively. According to the comparative analysis results of Coffalyser.Net, both SVs were confirmed to be heterozygous deletions (Fig. 1).

## Discussion

Sudden unexplained death (SUD) takes up a considerable part in overall sudden death cases, especially in adolescents and young adults. During the past decade, many channelopathy- and cardiomyopathy-associated SNVs have been identified in SUD studies by means of postmortem molecular autopsy [3–6], yet the number of cases that remain inconclusive is still high. The aim of this study was to re-analyze the exome data of 45 SUD cases according to the current ACMG guidelines and to search for potentially pathogenic SNVs and SVs.

Exome sequencing data was already available for 35 SUD cases, whereas additional 10 SUD cases were sequenced and analyzed for rare SNVs within the scope of this study. Following the current ACMG guidelines for variant interpretation [26], we re-analyzed and re-classified the SNVs in the 35 SUD cases in addition to the 10 newly sequenced SUD cases. A total of 14 pathogenic or likely pathogenic variants were identified in 10 (22.2%) of the 45 SUD cases. Out of these 10 cases, 1 individual (2.2%) carried a pathogenic variant in the cardiomyopathy-associated gene *DTNA* and 2 individuals (4.4%) carried 2 (1 pathogenic and 1 likely pathogenic) variants in the channelopathy-associated genes *SCN5A* and *TRDN*, respectively. Variants in the alpha-dystrobrevin encoding gene *DTNA* have been reported in patients with congenital heart disease and left ventricular non-compaction (LVNC) [41]. The *SCN5A* gene encodes the alpha subunit of the main cardiac sodium channel Na_v_1.5 and variants in this gene have been found to be causatively associated with BrS, long QT syndrome, cardiac conduction system dysfunction, and dilated cardiomyopathy [42]. Triadin-1, encoded by *TRDN*, is an important component of the calcium release unit in the sarcoplasmic reticulum of cardiac myocytes and a number of variants have been identified in patients with catecholaminergic polymorphic ventricular tachycardia (CPVT) [43].

Other studies already pointed out the importance of re-analysis of sequencing data, especially in patients with complex genetic diseases, as genomic databases are continuously updated and adjusted based on new findings in co-segregation studies and functional analyses [44]. Accordingly, when comparing our previously published data and the results presented in this study, some variants were interpreted differently and some new variants popped up. These changes are caused by the updated candidate gene list, a more stringent MAF as recommend by Tester et al. [45] and the assessment of pathogenicity based on the genome interpretation database VarSome that includes additional interpretation criteria, such as if the variant is located in a mutational hot spot and/or critical and well-established functional domain [35]. Nevertheless, many variants remain with uncertain significance emphasizing the importance of cautious interpretation, especially in cases without a clear phenotype and a complex genetic contribution. In addition, co-segregation analysis and functional assays are recommended to further evaluate the pathogenicity of identified variants.

The focus of this study was a genetic screening for SVs as potential contribution to the sudden unexpected death event. A total of 18 SVs were identified in 15 out of the 45 individuals, but only 2 (11.1%) were located on exons. The 2 exonic SVs were confirmed by MLPA as heterozygous deletions. The functional annotation of the 2 SVs was checked in several databases.

The 71-bp heterozygous deletion in *PDSS2* was previously identified as nsv4140011 by whole genome sequencing (WGS) in the gnomAD structural variants study [39]. However, neither validation information nor clinical assertion has been reported for this SV. According to the Database of Genomic Variants (DGV) [38], the deletion type was only observed once within 10,847 samples. Such a low frequency could meet the criteria for a pathogenic SV. The protein encoded by *PDSS2* is an enzyme that synthesizes the prenyl side chain of coenzyme Q_10_, which is one of the key elements in the respiratory chain. Previous research has revealed that individuals with primary coenzyme Q_10_ deficiency could have manifestations associated with multisystemic diseases, including encephalopathy and hypertrophic cardiomyopathy [46]. Therefore, we have reason to suspect SVs that alter the function or expression of this gene might be pathogenic variants. In addition, it is noteworthy that a missense mutation with uncertain significance in *RYR2* (p.(Ala3814Val)) has also been found in this individual. However, it is not clear what the biological effects of these genetic variants are and whether they may have contributed to the cause of death in this young girl.

The other 35 bp heterozygous deletion in *TRPM4* was reported to have conflicting interpretations of pathogenicity (benign, likely benign, or uncertain significance) according to the NCBI ClinVar database. As a calcium-activated ion channel encoding gene, *TRPM4* had already been extensively studied in a series of channelopathy-related reports, and some uncommon missense SNVs had been identified to be likely pathogenic in 20 out of 248 BrS patients and in 13 out of 330 SUD cases, respectively [47, 48]. One study had demonstrated in-frame deletions in individuals with cardiac conduction disturbances, but the deletions co-existed with other missense variants, which makes it hard to determine their real functional impact [49]. In our case, beside the heterozygous deletion in *TRPM4*, a pathogenic stop variant in the gene *LZTR1* (p.(Gln814*)) was identified. Variants in *LZTR1* are associated with Noonan syndrome, which is a genetic disorder that causes multiple congenital abnormalities and characteristic facial features that evolve with age [50]. Furthermore, a small portion of patients with Noonan syndrome were reported to show cardiovascular diseases, including atrial septal defects and hypertrophic cardiomyopathy [51].

It is worth noting that a large proportion of SVs identified in this study were located in the intergenic or intronic regions of our target genes and thus were of unknown significance. The evaluation of variants in these noncoding regions has always been challenging as the knowledge about their contribution to electrophysiological dysfunction is still very limited. Introns are usually considered to contribute to the control of gene expression if regulatory regions and noncoding functional RNA genes are affected [52–54]. A recent study combined the most extensive maps of CNVs in human populations and discovered that intronic losses are the most frequent CNVs in protein-coding genes [55]. Therefore, the significance of SVs identified in the intronic regions of our target genes might need to be carefully evaluated by functional studies. Moreover, a recent study has cross-referenced human transcriptome, epigenomic, and chromatin datasets to find causal genetic variants in noncoding regions that alter the functionality of transcription regulatory elements and target gene expression associated with atrial fibrillation (AF) [56]. With an improved ability to identify these genetic variants neglected by most previous studies, the pathogenic mechanism behind SUD might eventually be better explained by routine SNV and SV testing in suspected SUD cases.

There are some limitations in our current study. Since we only focused on 244 cardiac-related genes, variants outside these regions could not be identified. Besides, SVs longer than 1 Mbp were not included in our candidate list due to the difficulties in confirming the MPS result. In addition, functional studies would be required to further investigate the 2 identified SVs in *PDSS2* and *TRPM4* in order to verify their potential pathogenic role and contribution to the sudden death event of these 2 SUD cases. When several potential pathogenic variants are under consideration, it will be important to find out which of the detected variants contributed most to the sudden unexpected death event. In our study, family members were not available for co-segregation analyses. This would be necessary to determine the mode of inheritance and to identify other family members at risk for sudden cardiac death.

In conclusion, our study supports that SVs in cardiac disease-associated genes might be involved in some SUD cases. However, the functional interpretation of pathogenic SVs is complex and genetic evidence should be used cautiously in molecular diagnosis.

## Supplementary Information

Below is the link to the electronic supplementary material.

Fig. S1 (PDF 168 kb)

Table S1 (XLSX 15 kb)

Table S2 (XLSX 12 kb)

Table S3 (XLSX 9 kb)

Table S4 (XLSX 16 kb)

Table S5 (XLSX 12.5 kb)
