# Supplementary material for: Re-evaluation of single nucleotide variants and identification of structural variants in a cohort of 45 sudden unexplained death cases
Source: Int J Legal Med. 2021 Apr 25;135(4):1341–9. doi: 10.1007/s00414-021-02580-5 (PMC8205883; doi:10.1007/s00414-021-02580-5)
Supplement: Supplementary file 1 — (PDF 168 kb) [file 414_2021_2580_MOESM1_ESM.pdf]

**Re-evaluation of single nucleotide variants and identification of structural variants in a cohort of 45 sudden unexplained death cases**

Jacqueline Neubauer[1,*,°], Shouyu Wang[1,3,*], Giancarlo Russo[2], Cordula Haas[1]

[1]Zurich Institute of Forensic Medicine, University of Zurich, Zurich, Switzerland

[2]Functional Genomics Center Zurich (FGCZ), University of Zurich / ETH, Switzerland

[3]Department of Forensic Medicine, School of Basic Medical Sciences, Fudan University, Shanghai, China

*Shouyu Wang and Jacqueline Neubauer contributed equally to this work.

°Corresponding author:

Jacqueline Neubauer

ORCID-ID: 0000-0002-9472-7803

jacqueline.neubauer@irm.uzh.ch

A

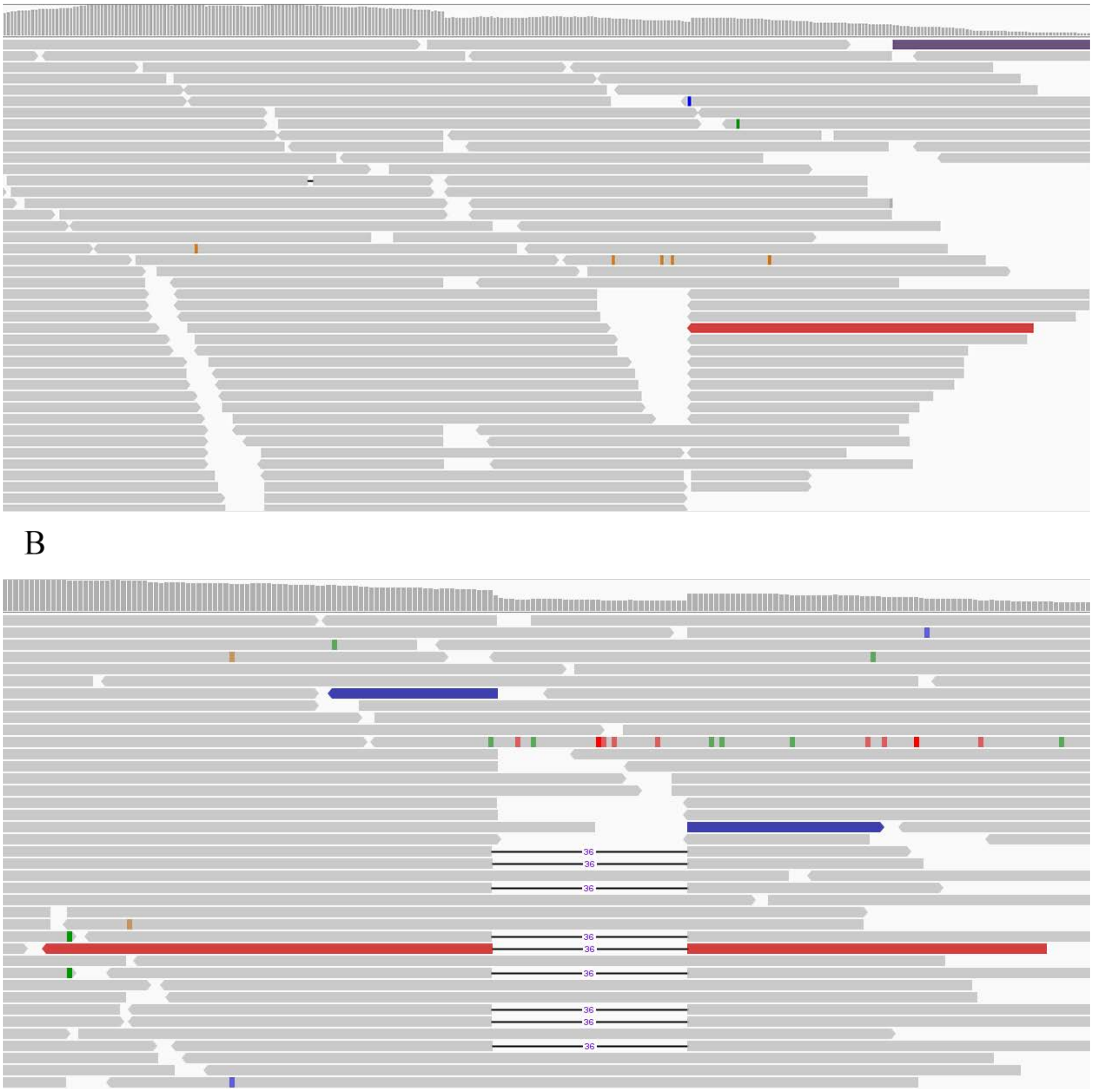


**Fig S1** Sequencing reads distribution of the SV candidates in (A) *PDSS2* (NM_020381.4: c.1009-4103_1009-4033del), and (B) *TRPM4* (NM_017636.4: c.1459_1494del, p.(Lys487_Leu498del)), respectively. The software IGV viewer v.2.4.16. was used for visualization.
